# Relationship between serum leptin, lipid metabolism, HbA1c, and renal function in individuals with type 2 diabetes mellitus and obesity and in individuals with type 2 diabetes mellitus without obesity

**DOI:** 10.1097/MD.0000000000045526

**Published:** 2025-11-14

**Authors:** Akintunde Akintayo Akinjinmi, Adebayo Adetola Amballi, Abdulbasit Opeyemi Abdulrahman, Nicholas Aderinto, Akinbobola Ayokunle Adeniyi, Ibukun Akinwunmi Akindele, Opeyemi Muili AbdulBasit, Emmanuel Ifeanyi Obeagu

**Affiliations:** a Department of Medical laboratory Science, Babcock University, Ilishan Remo, Ogun State, Nigeria; b Department of Chemical Pathology and Immunology, Olabisi Onabanjo University, Ago-Iwoye, Ogun, Nigeria; c Department of Medical Laboratory Science, College of Basic Health Sciences, Achievers University Owo, Ondo, Nigeria; d Department of Medicine and Surgery, Ladoke Akintola University of Technology, Ogbomoso, Nigeria; e Department of Medical Microbiology and Parasitology, University College Hospital, Ibadan, Nigeria; f Department of Biomedical and Laboratory Science, Africa University, Mutare, Zimbabwe.

**Keywords:** glycemic control, leptin, lipid metabolism, obesity, renal function, type 2 diabetes mellitus

## Abstract

Type 2 diabetes mellitus (T2DM) is a chronic metabolic disorder characterized by hyperglycemia, impaired lipid metabolism, and insulin resistance. In addition to the traditional risk factors, recent studies have suggested that adipose tissue-derived hormones such as leptin play a crucial role in the pathogenesis of T2DM and its related complications. However, the exact mechanism underlying the relationship between serum leptin levels and these comorbidities remains unclear. The study included 222 individuals, with 74 having T2DM and obesity, 74 having T2DM without obesity, and 74 in the control group. Serum levels of leptin, total cholesterol, triglycerides, high-density lipoprotein (HDL), low-density lipoprotein (LDL), and HbA1c were measured, and the glomerular filtration rate (GFR) was used as a measure of renal function. Serum leptin levels were significantly higher in individuals with T2DM and obesity compared to those without obesity (*P* < .05). Additionally, total cholesterol, triglycerides, and LDL were significantly higher in individuals with T2DM and obesity compared to those without obesity (*P* < .05). Serum leptin levels were positively correlated with total cholesterol, triglycerides, and LDL (*P* < .05) and negatively correlated with HDL (*P* < .01). The GFR was not significant when both groups were compared. Obesity has a negative impact on lipid profile and glycemic control in type 2 diabetes patients, which highlights the significance of considering the effect of obesity on the relationship between leptin and lipid profile in diabetic patients. These findings suggest that targeting obesity and leptin levels may be an effective strategy for improving lipid profiles and glycemic control in type 2 diabetes.

## 1. Introduction

Type 2 diabetes mellitus (T2DM) is a chronic metabolic disorder characterized by hyperglycemia, impaired lipid metabolism, and insulin resistance.^[1]^ The prevalence of T2DM has been steadily increasing worldwide, and it is a major public health concern due to its association with various comorbidities, including cardiovascular disease (CVD) and chronic kidney disease.^[2]^ In addition to the traditional risk factors, recent studies have suggested that adipose tissue-derived hormones such as leptin play a crucial role in the pathogenesis of T2DM and its related complications.^[3,4]^

Leptin is a hormone secreted by adipose tissue that regulates energy balance and appetite by suppressing hunger and promoting energy expenditure.^[5]^ It has been reported that serum leptin levels are elevated in individuals with obesity and T2DM.^[6,7]^ Furthermore, several studies have suggested that hyperleptinemia is associated with impaired lipid metabolism and renal dysfunction in T2DM.^[8,9]^ However, the exact mechanism underlying the relationship between serum leptin levels and these comorbidities remains unclear. The term “lipid metabolism” describes how the body breaks down and uses fats.^[10]^ Lipid metabolism is frequently dysregulated in people with T2DM and obesity, resulting in increased levels of blood lipids such as cholesterol and triglycerides.^[11]^ To explore the potential association between leptin levels and lipid metabolism in this population, the study examines the relationship between circulating leptin concentrations and lipid profile parameters among diabetic and nondiabetic individuals.

When evaluating glycemic control in diabetics, hemoglobin A1c (HbA1c), a marker of long-term blood glucose control, is frequently utilized.^[12]^ However, the act of losing weight has been identified as essential for managing T2DM and achieving remission.^[13]^ Weight loss may help overweight and obese T2DM patients have a better cardiovascular risk profile, including better glycemic and blood pressure control. The HbA1c level, which might indicate how well diabetes patients are managing their blood glucose, is also directly associated with the risk of diabetic complications.^[14]^ The goal of the study is to determine if leptin affects glycemic control and the onset or progression of T2DM in obese people by evaluating the association between serum leptin levels and HbA1c.

Renal function is the term used to describe how well the kidneys are functioning and their capacity to remove waste from the blood.^[15]^ Obesity and T2DM both increase the risk of renal problems, including diabetic nephropathy.^[16]^ In order to shed light on the potential influence of leptin on kidney health in this population, this study examines whether serum leptin levels are related to renal function in people with T2DM and obesity.

## 2. Methodology

A total of 222 individuals of both sexes age 18 to 65 years, were involved in this study. Test subjects were recruited from the diabetes clinic of Olabisi Onabanjo University Teaching Hospital, Sagamu while Control subjects were recruited from the patients at General outpatient clinic of the same health institution. The study is a cross-sectional study design.

### 2.1. Study patients

A total of 222 individuals of both sexes aged 18 to 65 years (74 obese individuals (aged 49.8 ± 10.5 years) and 74 non-obese individuals (aged 47.9 ± 9.6), both with type 2 diabetes mellitus, who consecutively visited the diabetes clinic of the Olabisi Onabanjo University Teaching Hospital, Sagamu, Ogun State, Nigeria, and 74 screened individuals (aged 48.3 ± 11.2 years) that do not have diabetes, were also recruited as control individuals from the patients at the general outpatient clinic of the same health institution for 3 months, T2DM was defined based on the history of patients taking oral hypoglycemic drugs or according to the classification of the American Diabetes Association as showing a fasting plasma glucose concentration >126 mg/dL.^[1]^ Individuals with diabetes were treated with oral hypoglycemic agents (metformin, n = 30, glibenclamide, n = 11). No patients received insulin therapy. None of the individuals suffered from significant renal, hepatic, or cardiovascular diseases. Diabetes lasted between 1 and 6 years (mean: 2.50 ± 1.44 years). Patients did not consume alcohol or perform heavy exercises for at least 1 week before the study.

The nondiabetic control group included 74 middle-aged and elderly non-obese people aged 48.3 ± 11.2 years who had received an annual health checkup. The following criteria were used to select the nondiabetic control individuals: No diabetes in their first-degree relatives, fasting plasma glucose level <110 mg/dL, hemoglobin A1c concentration <5.5%. Nondiabetic subjects with endocrine disease, significant renal or hepatic diseases, or those receiving medications that control glucose metabolism, hypertension, or hyperlipidemia were excluded from the study. Non-obesity was defined according to WHO criteria (BMI < 30 kg/m^2^).^[2]^

### 2.2. Anthropometric evaluation

Indicators of anthropometry such as height, weight, hip circumference, and waist circumference were measured while subjects were standing and dressed comfortably without shoes. The body mass index (BMI) was determined based on height and weight measurements in kilograms and centimeters, respectively.^[3]^ The hip circumference was measured at the largest standing horizontal circumference of the buttocks, and the waist circumference was measured at the lowest standing horizontal circumference between the lower margin of the rib cage and the iliac crest. The waist-to-hip ratio (WHR) was also determined by dividing hip circumference by waist circumference, which is the ratio of waist circumference to hip circumference in cm.^[3]^ Well-trained nutritionists measured these variables. The medical officer thoroughly examined the participants to determine their current state of health.

### 2.3. Analytical methods

Following an overnight fast of 10 to 12 hours, 10 mL of venous blood was collected from each participant. Samples were dispensed into appropriate collection tubes: fluoride-oxalate bottles for fasting plasma glucose, EDTA bottles for glycated hemoglobin (HbA1c), and serum-separating tubes for leptin, urea, creatinine, and lipid profile. After centrifugation, plasma and serum were immediately separated, aliquoted into plain bottles, and stored at −20°C until analysis.^[17–22]^

### 2.4. Biochemical analysis

Fasting plasma glucose was measured using the glucose oxidase enzymatic method.^[19]^ Glycated hemoglobin (HbA1c) was determined immunoturbidimetrically using a microparticle agglutination inhibition method.^[23]^ Lipid profile components including total cholesterol, triglycerides, HDL-C, and calculated LDL-C were analyzed using Randox test kits,^[21]^ with LDL cholesterol calculated using the Friedewald formula for samples with triglyceride concentrations below 400 mg/dL.

Urea and creatinine levels were assessed using the urease method and the Jaffe reaction, respectively.^[22]^ Serum leptin concentrations were determined using a human-specific enzyme-linked immunosorbent assay (ELISA) kit (BioVendor Laboratory Medicine, Inc., GmbH, Brno, Czech Republic).^[20]^ Quality control procedures were followed, and all sera were diluted 1:3 with a diluting buffer prior to leptin analysis. The intra- and inter-assay coefficients of variation for leptin were both below 5%. All assays were conducted in accordance with the manufacturers’ instructions.

### 2.5. Ethical approval

The Olabisi Onabanjo University Teaching Hospital’s Health Research Ethics Committee evaluated and approved the study (NHREC/28/11/2017), and all individuals provided written informed consent following a description of the method.

### 2.6. Statistical analysis

Statistical analysis of the data was done using Statistical Package for Social Sciences (SPSS), Version 19 (IBM Corp., Armonk). Descriptive statistics and cross-tabulation were used to show the relationships between variables. The statistical significance of the difference in proportion was evaluated using analysis of variance, post hoc test and the *Z*-test was utilized as a statistical measure for continuous variables. Pearson Correlation was used to find the correlation between the biophysical and biochemical parameters. The level of statistical significance for all tests was set at *P* < .05. Stratifying the analysis by relevant demographic and clinical characteristics (e.g., age group, obesity status) allows for the examination of associations within homogeneous subgroups, thereby controlling for potential confounding by these factors. Also, matching individuals with T2DM with and without obesity based on key demographic and clinical characteristics help control for confounding by these variables.

## 3. Result

Table 1 shows the mean values and standard deviations of the biophysical measurements of diabetic non-obese subjects and diabetic obese subjects. There was no statistically significant difference (*P* > .05) between the mean age of diabetic non-obese (47.9 ± 9.6) compared with diabetic obese (49.8 ± 10.5). There was a statistically significant difference (*P* < .05) between the BMI of diabetic non-obese (23.4 ± 1.6) compared with diabetic obese (33.7 ± 3.7). There was also a statistically significant difference (*P* < .05) between the weight (kg) of diabetic non-obese (66.7 ± 8.2) compared with diabetic obese (90.1 ± 12.7). The waist-hip ratio of the diabetic non-obese subjects (0.85 ± 0.05) compared with diabetic obese (0.87 ± 0.09) showed a statistically significant difference (*P* < .05).

**Table 1:** Biophysical measurements of DM nonobese and DM obese (mean ± SD).

Parameters	Diabetic nonobese, mean ± SD	DM obese, mean ± SD	*Z*-test	*P*-value
Age (yr)	47.9 ± 9.6	49.8 ± 10.5	−0.951	.524
Weight (kg)	66.7 ± 8.2	90.1 ± 12.7	−13.171	.000***
BMI (kg/m^2^)	23.4 ± 1.6	33.7 ± 3.7	−21.008	.000***
WHR	0.85 ± 0.05	0.87 ± 0.09	−4.311	.015*

BMI = body mass index, DM = diabetes mellitus, SD = standard deviation, WHR = waist hip ratio.

Level of significance, *P* <.05.

* Statistically significant (*P* < .05).

*** Statistically significant (*P* < .0001).

Table 2 shows the mean values and standard deviations of the Biophysical measurements of diabetic non-obese subjects and control. There were no statistically significant differences (*P* > .05) between the mean age of diabetic non-obese (47.9 ± 9.6) compared with controls (48.3 ± 11.2) (*P* > .05), BMI of diabetic non-obese (23.4 ± 1.6) compared with controls (23.3 ± 1.6) (*P* > .05), weight (kg) of diabetic non-obese (66.7 ± 8.2) compared with controls (65.5 ± 8.2) (*P* > .05). The waist-hip ratio of the diabetic non-obese subjects (0.85 ± 0.05) compared with controls (0.85 ± 0.06) also showed no statistically significant difference (*P* > .05).

**Table 2:** Biophysical measurements of DM nonobese, DM obese, and control (mean ± SD).

Paramters	Diabetic nonobese, mean ± SD	Control, mean ± SD	*Z*-test	*P*-value
Age (yr)	47.9 ± 9.6	48.3 ± 11.2	7.463	.579
Weight (kg)	66.7 ± 8.2	65.5 ± 8.2	0.893	.373
BMI (kg/m^2^)	23.4 ± 1.6	23.3 ± 1.6	0.343	.732
WHR	0.85 ± 0.05	0.85 ± 0.06	0.869	.339

BMI = body mass index, DM = diabetes mellitus, SD = standard deviation, WHR = waist hip ratio.

Level of significance, *P* <.05.

Table 3 shows the mean values and standard deviations of the Biophysical measurements of Diabetic obese subjects and controls. There was no statistically significant difference (*P* > .05) between the mean age of diabetic obese (49.8 ± 10.5) compared with controls (48.3 ± 11.2). There was a statistically significant difference (*P* < .05) between the BMI of diabetic obese (33.7 ± 3.7) compared with controls (23.3 ± 1.6). There was also a statistically significant difference (*P* < .05) between the weight (kg) of diabetic obese (90.1 ± 12.7) compared with controls (65.5 ± 8.2). The waist-hip ratio of the diabetic obese subjects (0.87 ± 0.09) to controls (0.85 ± 0.06) showed a statistically significant difference (*P* < .05).

**Table 3:** Biophysical measurements of DM obese and control (mean ± SD).

Parameters	Diabetic obese, mean ± SD	Control, mean ± SD	*Z*-test	*P*-value
Age (yr)	49.8 ± 10.5	48.3 ± 11.2	3.329	.233
Weight (kg)	90.1 ± 12.7	65.5 ± 8.2	13.673	.000***
BMI	33.7 ± 3.7	23.3 ± 1.6	21.193	.000***
WHR	0.87 ± 0.09	0.85 ± 0.06	2.705	.000***

BMI = body mass index, DM = diabetes mellitus, SD = standard deviation, WHR = waist hip ratio.

Level of significance *P* <.05.

*** Statistically significant (*P*<.0001).

Table 4 shows the mean values and standard deviations of test and control subjects’ biophysical measurements. There were no statistically significant differences (*P* > .05) between the mean ages of diabetic non-obese subjects (47.9 ± 9.6) and diabetic obese subjects (49.8 ± 10.5) compared with controls (48.3 ± 11.2). There were statistically significant differences (*P* < .05) between the BMI of diabetic non-obese subjects (23.4 ± 1.6) and diabetic obese subjects (33.7 ± 3.7) compared with controls (23.3 ± 1.6). The waist-hip ratio of the diabetic non-obese subjects (0.85 ± 0.05) and diabetic obese subjects (0.87 ± 0.09) compared with controls (0.85 ± 0.06) showed a statistically significant difference (*P* < .05).

**Table 4:** Biophysical measurements of DM nonobese, DM obese, and control (mean ± SD) using analysis of variance.

Parameters	Diabetic nonobese, mean ± SD	DM obese, mean ± SD	Control, mean ± SD	*F*-value	*P*-value
Age	47.9 ± 9.6	49.8 ± 10.5	48.3 ± 11.2	54.151	.472
Weight (kg)	66.7 ± 8.2	90.1 ± 12.7	65.5 ± 8.2	28.925	.000***
BMI	23.4 ± 1.6	33.7 ± 3.7	23.3 ± 1.6	6.157	.000***
WHR	0.85 ± 0.05	0.87 ± 0.09	0.85 ± 0.06	4.311	.015*

BMI = body mass index, DM = diabetes mellitus, SD = standard deviation, WHR = waist hip ratio.

Level of significance *P* <.05.

* Statistically significant (*P*<.05).

*** Statistically significant (*P* < .0001).

Table 5 shows the mean values and comparisons of the biochemical parameters of diabetic non-obese subjects and diabetic obese subjects. There was no statistical difference (*P* > .05) between the mean Triglyceride (TG) levels of the diabetic non-obese subjects (102.0 ± 41.7) compared with diabetic obese (105.8 ± 48.6). There were statistically significant differences (*P* < .05) between the mean values of HbA1c of diabetic non-obese subjects (6.7 ± 1.1) compared with diabetic obese subjects (8.0 ± 1.9), leptin of diabetic non-obese subjects (6.1 ± 5.1) compared with diabetic obese subjects (8.1 ± 6.5) (*P* < .05); total cholesterol (TCHOL) of diabetic non-obese subjects (192.1 ± 33.6) compared with diabetic obese subjects (221.9 ± 52.0); high-density lipoprotein (HDL) of diabetic non-obese subjects (54.3 ± 23.7) compared with diabetic obese subjects (45.1 ± 17.0); low-density lipoprotein (LDL) of diabetic non-obese subjects (103.0 ± 31.2) and diabetic obese (155.3 ± 56.0).

**Table 5:** Biochemical parameters of DM nonobese and DM obese subjects (mean ± SD).

Parameters	Diabetic nonobese, mean ± SD	Diabetic obese, mean ± SD	*Z*-test	*P*-value
HbA1c (%)	6.7 ± 1.1	8.0 ± 1.9	−4.974.	.000***
Leptin (1.18–3.82 ng/mL)	6.1 ± 5.1	8.1 ± 6.5	−2.056	.042*
TCHOL (130–230 mg/dL)	192.1 ± 33.6	221.9 ± 52.0	−4.143	.000***
TRIG (30–170 mg/dL)	102.0 ± 41.7	105.8 ± 48.6	−.518	.605
HDL (30–70 mg/dL)	54.3 ± 23.7	45.1 ± 17.0	2.710	.008**
LDL (100–159 mg/dL)	103.0 ± 31.2	155.3 ± 56.0	−7.020	.000***
Urea (0–50mg/dL)	31.3 ± 9.8	33.4 ± 10.9	1.724	.087
Creat (0.5–1.2 mg/dL)	.85 ± 2.3	.87 ± 11.9	−1.072	.285
FPG (mg/dL)	125.8 ± 45.6	128.4 ± 44.0	−352	.725
GFR (mL/min)	111.1 ± 25.00	110.5 ± 39.2	−352	.725

DM = diabetes mellitus, FPG = fasting plasma glucose, GFR = glomerular filtration rate, HbA1c = glycated hemoglobin, HDL = high density lipoprotein, LDL = low density lipoprotein, TCHOL = total cholesterol, TRIG = triglyceride.

Level of significance *P*<.05.

* Statistically significant (*P*<.05).

** Statistically significant (*P*<.001).

*** Statistically significant (*P* < .0001).

Table 6 shows the mean values and comparisons of the biochemical parameters of diabetic non-obese subjects and controls. There was no statistically significant difference between the mean Leptin of diabetic non-obese subjects (6.1 ± 5.1) compared with controls (5.3 ± 5.9), HDL of diabetic non-obese subjects (54.3 ± 23.7) compared with controls (51.7 ± 16.2). There were statistically significant differences (*P* < .05) between the mean values of HbA1c of diabetic non-obese subjects (6.7 ± 1.1) compared with control (4.9 ± .73); total cholesterol (TCHOL) of diabetic non-obese subjects (192.1 ± 33.6) compared with controls (204.9 ± 43.9); LDL of diabetic non-obese subjects (103.0 ± 31.2) compared with control subjects (133.5 ± 45.4); FPG of diabetic non-obese subjects (125.8 ± 45.6) compared with control subjects (88.7 ± 4.6) (*P* < .05).

**Table 6:** Biochemical parameters of DM nonobese and control subjects (mean ± SD).

Parameters	Diabetic nonobese, mean ± SD	Control, mean ± SD	*Z*-test	*P*-values
HbA1c (%)	6.7 ± 1.1	4.9±.73	11.094	.000***
Leptin (1.18–3.82 ng/mL)	6.1 ± 5.1	5.3 ± 5.9	0.869	.386
TCHOL (130–230 mg/dL)	192.1 ± 33.6	204.9 ± 43.9	−1.993	.048*
TRIG (30–170 mg/dL)	102.0 ± 41.7	93.2 ± 36.3	1.366	.174
HDL (30–70 mg/dL)	54.3 ± 23.7	51.7 ± 16.2	0.794	.429
LDL (100–159 mg/dL)	103.0 ± 31.2	133.5 ± 45.4	−4.757	.000***
Urea (0–50 mg/dL)	31.3 ± 9.8	30.2 ± 7.1	1.541	.126
Creat (0.5–1.2 mg/dL)	0.85 ± 2.3	0.77 ±.22	−2.547	.012*
FPG (mg/dL)	125.8 ± 45.6	88.7 ± 4.6	6.156	.000***
GFR (mL/min)	111.1 ± 25.00	113.0 ± 31.4	−2.085	.776

DM = diabetes mellitus, FPG = fasting plasma glucose, GFR = glomerular filtration rate, HbA1c = glycated hemoglobin, HDL = high density lipoprotein, LDL = low density lipoprotein, TCHOL = total cholesterol, TRIG = triglyceride.

Level of significance *P*<.05.

* Statistically significant (*P* < .05).

*** Statistically significant (*P* < .0001).

Table 7 shows the mean values and comparisons of the biochemical parameters of obese diabetic subjects and controls. There was no statistically significant difference between the mean triglyceride of diabetic obese subjects (105.8 ± 48.6) and controls (93.2 ± 36.3). There were statistically significant differences (*P* < .05) between the mean values of HbA1c of diabetic obese subjects (8.0 ± 1.9) and control (4.9 ± 0.73); Leptin of diabetic obese subjects (8.1 ± 6.5) and controls (5.3 ± 5.9); total cholesterol (TCHOL) of diabetic obese subjects (221.9 ± 52.0) and controls subjects (204.9 ± 43.9); HDL of diabetic obese subjects (45.1 ± 17.0) and controls (51.7 ± 16.2); LDL of diabetic obese subjects (155.3 ± 56.0) and control subjects (133.5 ± 45.4); FPG of diabetic obese subjects (128.4 ± 44.0) and control subjects (88.7 ± 4.6).

**Table 7:** Biochemical parameters of DM obese and control subjects (mean ± SD).

Parameters	Diabetic obese, mean ± SD	Control, mean ± SD	*Z*-test	*P*-value
HbA1c (%)	8.0 ± 1.9	4.9±.73	12.699	.000***
Leptin (1.18–3.82 ng/mL)	8.1 ± 6.5	5.3 ± 5.9	2.705	.008**
TCHOL (130–230 mg/dL)	221.9 ± 52.0	204.9 ± 43.9	2.149	.033*
TRIG (30–170 mg/dL)	105.8 ± 48.6	93.2 ± 36.3	1.792	.075
HDL (30–70 mg/dL)	45.1 ± 17.0	51.7 ± 16.2	−2.392	.018*
LDL (100–159 mg/dL)	155.3 ± 56.0	133.5 ± 45.4	2.604	.010*
UREA (0–50 mg/dL)	33.4 ± 10.9	30.2 ± 7.1	−0.513	.609
CREAT (0.5–1.2 mg/dL)	0.87 ± 11.9	0.77 ±.22	1.013	.313
FPG (mg/dL)	128.4 ± 44.0	88.7 ± 4.6	6.769	.000***
GFR (mL/min)	110.5 ± 39.2	113.0 ± 31.4	0.097	.923

DM = diabetes mellitus, FPG = fasting plasma glucose, GFR = glomerular filtration rate, HbA1c = glycated hemoglobin, HDL = high density lipoprotein, LDL = low density lipoprotein, TCHOL = total cholesterol, TRIG = triglyceride.

Level of significance *P*<.05.

* Statistically significant (*P* < .05).

** Statistically significant (*P* <.001).

*** Statistically significant (*P* <.0001).

Table 8 shows the comparison of the biochemical parameters of DM non-obese, DM Obese and control (Mean ± SD) using analysis of variance. There was a statistically significant difference in the mean concentration of HbA1c, leptin, total cholesterol (TCHOL), HDL, LDL and FPG between the 3 groups (DM non-obese, DM obese and control) with *P*-value (<.05). While the there was no statistically significant difference in the mean concentration of Triglyceride, Urea, Creatinine and glomerular filtration rate (GFR) between the 3 groups (DM non-obese, DM obese and control) with *P*-value (>0.05).

**Table 8:** Biochemical parameters of DM nonobese, DM obese, and control (mean ± SD) using analysis of variance.

Parameters	DM nonobese, mean ± SD	DM obese, mean ± SD	Control, mean ± SD	*F*-value	*P*-value
HbA1c (%)	6.7 ± 1.1	8.0 ± 1.9	4.9±.73	93.185	.000***
Leptin (1.18–3.82 ng/mL)	6.1 ± 5.1	8.1 ± 6.5	5.3 ± 5.9	4.365	.014*
TCHOL (130–230 mg/dL)	192.1 ± 33.6	221.9 ± 52.0	204.9 ± 43.9	8.619	.000***
TRIG (30–170 mg/dL)	102.0 ± 41.7	105.8 ± 48.6	93.2 ± 36.3	1.718	.182
HDL (30–70 mg/dL)	54.3 ± 23.7	45.1 ± 17.0	51.7 ± 16.2	4.458	.013*
LDL (100–159 mg/dL)	103.0 ± 31.2	155.3 ± 56.0	133.5 ± 45.4	24.820	.000***
UREA (0–50 mg/dL)	31.3 ± 9.8	33.4 ± 10.9	30.2 ± 7.1	1.944	.146
Creat (0.5–1.2 mg/dL)	0.85 ± 2.3	0.87 ± 11.9	0.77 ±.22	1.089	.336
FPG (mg/dL)	125.8 ± 45.6	128.4 ± 44.0	88.7 ± 4.6	23.669	.000***
GFR (mL/min)	111.1 ± 25.00	110.5 ± 39.2	113.0 ± 31.4	1.718	.284

DM = diabetes mellitus, FPG = fasting plasma glucose, GFR = glomerular filtration rate, HbA1c = glycated hemoglobin, HDL = high density lipoprotein, LDL = low density lipoprotein, TCHOL = total cholesterol, TRIG = triglyceride.

Level of significance *P*<.05.

* Statistically significant (*P* < .05).

*** Statistically significant (*P* < .0001).

Table 9 shows the comparison of the measured parameters of DM non-obese, DM Obese and control (Mean ± SD) using post hoc analysis. There was no significant difference in the level of FPG between DM non-obese and DM obese (*P* > .05), there was a significant difference between the level of FPG between control and DM non-obese (*P* < .05), there was also a significant difference in the level of FPG between control and DM obese (*P* < .05). There was significant difference in the level of HbA1c between DM non-obese and DM obese (*P* < .05), there was a significant difference between the level of HbA1c between control and DM non-obese (*P* < .05), there was also a significant difference in the level of HbA1c between control and DM obese (*P* < .05). There was no significant difference in the level of leptin between DM non-obese and DM obese (*P* > .05), there was no significant difference between the level of FPG between control and DM non-obese (*P* > .05), while there was a significant difference in the level of leptin between control and DM obese (*P* < .05). There was a significant difference in the level of total cholesterol (TCHOL) between DM non-obese and DM obese (*P* < .05), there was no significant difference between the level of total cholesterol (TCHOL) between control and DM non-obese (*P* > .05), there was also a significant difference in the level of total cholesterol (TCHOL) between control and DM obese (*P* < .05). There was no significant difference in the level of Triglyceride between DM non-obese and DM obese (*P* > .05), there was a significant difference between the level of Triglyceride between control and DM non-obese (*P* > .05), there was also a significant difference in the level of Triglyceride between control and DM obese (*P* > .05). There was a significant difference in the level of HDL between DM non-obese and DM obese (*P* < .05), there was no significant difference between the level of HDL between control and DM non-obese (*P* > .05), there was also no significant difference in the level of HDL between control and DM obese (*P* > .05). There was a significant difference in the level of LDL between DM non-obese and DM obese (*P* < .05), there was a significant difference between the level of LDL between control and DM non-obese (*P* < .05), there was also a significant difference in the level of LDL between control and DM obese (*P* < .05). There was no significant difference in the level of urea between DM non-obese and DM obese (*P* > .05), there was no significant difference between the level of urea between control and DM non-obese (*P* > .05), there was also no significant difference in the level of urea between control and DM obese (*P* > .05). There was no significant difference in the level of creatinine between DM non-obese and DM obese (*P* > .05), there was no significant difference between the level of creatinine between control and DM non-obese (*P* > .05), there was also no significant difference in the level of creatinine between control and DM obese (*P* > .05). There was no significant difference in the level of GFR between DM non-obese and DM obese (*P* > .05), there was no significant difference between the level of GFR between control and DM non-obese (*P* > .05), there was also no significant difference in the level of GFR between control and DM obese (*P* > .05).

**Table 9:** Measured parameters of DM nonobese, DM obese, and control (mean ± SD) using post-hoc analysis.

Parameters	(I) Subject	(J) Subject	*P*-value
FPG (mg/dL)	DM nonobese	DM obese	.915
		Control	.000***
	DM obese	DM nonobese	.915
		Control	.000***
	Control	DM nonobese	.000***
		DM obese	.000***
HbA1c (%)	DM nonobese	DM obese	.000***
		Control	.000***
	DM obese	DM nonobese	.000***
		Control	.000***
	Control	DM nonobese	.000***
		DM obese	.000***
Leptin (ng/mL)	DM nonobese	DM obese	.102
		Control	.694
	DM obese	DM nonobese	.102
		Control	.013*
	Control	DM nonobese	.694
		DM obese	.013*
TCHOL (mg/dL)	DM nonobese	DM obese	.000***
		Control	.179
	DM obese	DM nonobese	.000***
		Control	.050*
	Control	DM nonobese	.179
		DM obese	.050*
TRIG (mg/dL)	DM nonobese	DM obese	.846
		Control	.421
	DM obese	DM nonobese	.846
		Control	.169
	Control	DM nonobese	.421
		DM obese	.169
HDL (mg/dL)	DM nonobese	DM obese	.011*
		Control	.681
	DM obese	DM nonobese	.011*
		Control	.100
	Control	DM nonobese	.681
		DM obese	.100
LDL (mg/dL)	DM nonobese	DM obese	.000***
		Control	.000***
	DM obese	DM nonobese	.000***
		Control	.011*
	Control	DM nonobese	.000***
		DM obese	.011*
UREA (mg/dL)	DM nonobese	DM obese	.141
		Control	.342
	DM obese	DM nonobese	.141
		Control	.871
	Control	DM nonobese	.342
		DM obese	.871
Creat (mg/dL)	DM nonobese	DM obese	.389
		Control	.997
	DM obese	DM nonobese	.389
		Control	.431
	Control	DM nonobese	.997
		DM obese	.431
GFR (mL/min)	DM nonobese	DM obese	.725
		Control	.776
	DM obese	DM nonobese	.725
		Control	.923
	Control	DM nonobese	.776
		DM obese	.923

DM = diabetes mellitus, FPG = fasting plasma glucose, GFR = glomerular filtration rate, HbA1c = glycated hemoglobin, HDL = high density lipoprotein, LDL = low density lipoprotein, TCHOL = total cholesterol, TRIG = triglyceride.

Level of significance *P* < .05.

* Statistically significant (*P* < .05).

*** Statistically significant (*P* < .0001).

Table 10 shows the Pearson correlation coefficient between leptin and biophysical parameters in the DM subjects (diabetic non-obese, diabetic obese) and controls. There was no significant association between leptin level against any of the biophysical parameters of the DM non-obese patients and controls. Leptin was significantly correlated with weight (*R* = 0.194, *P* < .05) in DM obese patients. Leptin was significantly correlated with BMI (*R* = 0.115, *P* < .05) in DM obese patients. Waist-hip ratio was also found significantly correlated with Leptin (*R* = 0.488, *P* < .05) in DM obese patients.

**Table 10:** Correlations between leptin and biophysical parameters in the diabetic mellitus subjects (diabetic nonobese, diabetic obese) and control.

ID	Leptin	Biophysical	n	*r* value	*P* value
Diabetic nonobese		Weight	74	0.024	.837
		Height	74	0.35	.766
		BMI	74	0.077	.516
		Waist–hip ratio	74	0.082	.264
Diabetic obese		Weight	74	0.194	.001*
		Height	74	0.081	.491
		BMI	74	0.115	.000*
		Waist–hip ratio	74	0.488	.000*
Control		Weight	74	0.160	.173
		Height	74	0.063	.594
		BMI	74	0.103	.384
		Waist–hip ratio	74	0.082	.489

BMI = body mass index.

* Correlation is significant at the .05 level (2-tailed).

Table 11 shows the Pearson correlation coefficient between leptin and lipid profile in the DM subjects (diabetic non-obese, diabetic obese) and controls. There was no significant association between leptin level and the lipid profile of the DM non-obese patients and controls. Leptin was significantly correlated with Triglyceride (*R* = 0.314, *P* < .05) in DM obese patients. Leptin was also significantly correlated with LDL (*R* = 0.115, *P* < .05) in DM obese patients. Leptin was also found to have no significant correlation with total Cholesterol (*R* = 0.118, *P* > .05) and HDL (*R* = 0.123, *P* > .05) in DM obese patients.

**Table 11:** Correlations between leptin and lipid profile in the DM subjects (diabetic nonobese, diabetic obese) and control.

Group	Leptin	Biochemical	n	*r*-value	*P*-value
Diabetic nonobese	–	TCHOL (130–230 mg/dL)	74	0.094	.425
	–	TRIG (30–170 mg/dL)	74	0.147	.213
	–	HDL (30–70 mg/dL)	74	−0.071	.293
	–	LDL (100–159 mg/dL) HbA1c%	74 74	0.105 0.054	.603 .073
Diabetic obese	–	TCHOL (130–230 mg/dL)	74	0.118	.318
	–	TRIG (30–170 mg/dL)	74	0.314	.005**
	–	HDL (30–70 mg/dL)	74	−0.099	.296
	–	LDL (100–159 mg/dL) HbA1c%	74 74	0.123 0.027	.007** .819
Control	–	TCHOL (130–230 mg/dL)	74	0.173	.141
	–	TRIG (30–170 mg/dL)	74	0.024	.839
	–	HDL (30–70 mg/dL)	74	0.101	.363
	–	LDL (100–159 mg/dL) HbA1c%	74	0.107 0.008	.363 .945

DM = diabetes mellitus, HDL = high density lipoprotein, LDL = low density lipoprotein, n = sample size, *r*-value = Pearson correlation coefficient, TCHOL = total cholesterol, TRIG = triglyceride.

** Correlation is significant at the .01 level (2-tailed).

Table 12 shows the Pearson correlation coefficient between Leptin and Urea, Creatinine and eGFR of DM subjects (diabetic non-obese, diabetic obese) and controls. There was no significant association between Leptin level and the Urea, Creatinine and eGFR of the DM subjects and controls.

**Table 12:** Correlations between leptin and urea, creatinine and estimated GFR in the DM subjects (diabetic nonobese, diabetic obese) and control.

Group	Leptin	Biochemical	n	*r*-value	*P*-value
Diabetic nonobese	–	Urea (0–50 mg/dL)	74	0.052	.268
	–	Creat (0.5–1.2 mg/dL)	74	0.072	.164
	–	GFR (mL/min)	74	−0.099	.739
Diabetic obese	–	Urea (0–50 mg/dL)	74	0.122	.299
	–	Creat (0.5–1.2 mg/dL)	74	0.170	.551
	–	GFR (mL/min)	74	−0.121	.939
Control	–	Urea (0–50 mg/dL)	74	0.072	.242
	–	Creat (0.5–1.2mg/dL)	74	0.131	.267
	–	GFR (mL/min)	74	−0.039	.304

Creat = creatinine, DM = diabetes mellitus, GFR = glomerular filtration rate, n = sample size, *r*-value = Pearson correlation coefficient.

## 4. Discussion

This study aims to investigate the relationship between serum leptin, lipid metabolism, HbA1c, and renal function in individuals with T2DM and obesity and individuals with T2DM without obesity. We hypothesize that serum leptin levels are associated with impaired lipid metabolism and renal function in individuals with T2DM and that this association may be influenced by the presence of obesity. The purpose of exploring the study is to reveal specific biomarkers that indicate disease severity, progression, and risk of complications in T2DM patients with and without obesity. For example, it may identify elevated serum leptin levels as a predictor of renal dysfunction in obese T2DM patients, highlighting leptin as a potential therapeutic target. More so, understanding how these factors interact can help stratify patients into risk categories based on their metabolic profile. This stratification enables physicians to priorities interventions for high-risk individuals who are more likely to experience complications such as cardiovascular disease and diabetic nephropathy. It is also believed that the study will guide personalized treatment approaches. For instance, it may suggest that T2DM patients with obesity and elevated leptin levels may benefit from interventions targeting leptin signaling to improve metabolic control and prevent renal complications. Findings from the study can inform the development of clinical guidelines and interventions tailored to specific patient subgroups. For example, it may prompt the inclusion of leptin measurement in routine clinical assessments for obese T2DM patients to better assess their risk of renal impairment and guide treatment decisions. However, the primary finding of the research is that the biomarkers are significantly higher in T2DM and obese individuals, while the secondary finding suggests that obesity has a negative impact on lipid profile and glycemic control in type 2 diabetes patients.

The study examine the profiles of blood leptin, lipids, HbA1C, and renal function in type 2 diabetes mellitus patients, with a particular emphasis on differences between non-obese and obese patients. To achieve this objective, a sample of 222 individuals was recruited, comprising 74 individuals diagnosed with type 2 diabetes mellitus but not classified as obese, 74 individuals classified as obese, and 74 healthy individuals serving as control subjects. The results of our study provide valuable insights into the complex relationship between obesity and type 2 diabetes. Our findings demonstrate the need for comprehensive evaluations of these metabolic and biochemical markers in type 2 diabetes patients, particularly with respect to differences between non-obese and obese individuals. These insights are crucial for the effective clinical management of type 2 diabetes, a debilitating and increasingly prevalent chronic condition. Based on the results of our study, a significant association has been established between obesity and type 2 diabetes in the studied population. Our findings, obtained through extensive data analysis and statistical evaluation, indicate that a higher BMI and waist-hip ratio are significantly associated with an increased risk of developing type 2 diabetes. This conclusion was drawn by comparing the differences between diabetic obese patients, diabetic non-obese patients, and a control group. These results have important implications for the early identification and management of type 2 diabetes and highlight the need for further research to better understand the underlying mechanisms linking obesity and type 2 diabetes.

From the findings on various physical measurements of this study on diabetic individuals who are not obese and those who are obese, it can be indicated that the mean age was not significantly different, suggesting age similarity between the groups of individuals. However, the BMI, which indicates weight relative to height, was significantly higher in diabetic obese individuals compared to diabetic non-obese individuals, suggesting that obese individuals had a higher proportion of body weight relative to their height. The findings of the study are similar to those reported by.^[29]^ The study also revealed that the weight (in kg/m^2^) was significantly higher in diabetic obese individuals compared to diabetic non-obese individuals, indicating a higher overall body weight in the obese group, which was consistent with the findings of.^[30]^ More so, the waist-hip ratio (WHR), which assesses fat distribution, was significantly different between the 2 groups, with diabetic obese individuals having a higher ratio compared to diabetic non-obese individuals. This indicates that obese individuals had more fat around their waists relative to their hips compared to non-obese individuals and is similar to the report by.^[31]^ It can be observed from this study that while age did not differ significantly between diabetic non-obese and diabetic obese individuals, there were significant differences in BMI, weight, and WHR, highlighting the impact of obesity on various physical measurements among diabetic individuals.

Our study’s results align with a considerable body of evidence that has established a strong association between obesity and type 2 diabetes. For instance, a systematic review and meta-analysis by Galicia-Garcia et al found that an elevated BMI was significantly associated with an increased risk of type 2 diabetes.^[1]^ Similarly, a study by Huang et al indicated that obesity, as evidenced by a high waist-hip ratio, was correlated with a heightened risk of type 2 diabetes.^[2]^ While obesity is a known risk factor for type 2 diabetes, several other factors, such as genetic predisposition, lifestyle habits, and other metabolic and hormonal imbalances, may also contribute to the development of the disease. Additionally, the precise mechanisms by which obesity contributes to the onset of type 2 diabetes remain to be fully understood and require further investigation.

In light of our study results, it is imperative to continue exploring the intricate relationship between demographic, anthropometric, metabolic, and hormonal factors in the etiology and progression of type 2 diabetes, particularly in populations with a heightened risk of obesity. Further research is essential to deepening our understanding of the underlying mechanisms of type 2 diabetes and to designing and implementing effective strategies for its prevention and management. Given the increasing prevalence of both obesity and type 2 diabetes, addressing this pressing public health challenge is of utmost importance. By conducting robust and comprehensive research, we can understand the interplay between these factors and develop targeted and effective interventions to reduce the burden of type 2 diabetes in affected populations. Furthermore, our results showed that obesity could have a negative impact on lipid profiles and glycemic control in these patients. Specifically, we found that while triglyceride levels did not differ significantly between the 2 groups, the obese group had significantly higher levels of HbA1c, leptin, total cholesterol, HDL, and LDL compared to the non-obese group. This indicates that obesity may contribute to the development of metabolic abnormalities in type 2 diabetes mellitus.

These findings are consistent with previous studies showing a positive correlation between obesity and HbA1c levels in type 2 diabetes mellitus patients.^[3–5]^ Similarly, research has reported elevated levels of total cholesterol and LDL in obese patients with type 2 diabetes mellitus.^[4]^ The higher levels of leptin in the obese group in our study also agree with prior studies that have established a connection between obesity and increased leptin levels. In addition, our results indicated that obese individuals with type 2 diabetes exhibited elevated HbA1c, leptin, total cholesterol, HDL, LDL, and fasting plasma glucose compared to non-obese individuals with the same condition. This difference was statistically significant (*P* > .05). The findings of our study concur with previous studies that have reported the negative impact of obesity on glycemic control and lipid profiles in type 2 diabetes patients.^[6,7]^ A study by Sherwani et al reported that obesity was associated with higher levels of HbA1c and fasting plasma glucose in type 2 diabetes patients, which supports our findings.^[8]^ Another study by Hussain et al found that obesity was linked to elevated levels of total cholesterol, LDL, and triglycerides, consistent with our results regarding the impact of obesity on the lipid profile in type 2 diabetes patients.^[9]^ However, in contrast to our findings, Amidu et al reported no significant difference in HbA1c, triglycerides, or fasting plasma glucose levels between obese and non-obese type 2 diabetes patients.^[24]^ This discrepancy may be due to differences in study populations, sample sizes, or methodologies used in the studies.

The novelty of the research relies on its ability to efficiently examine the interconnection of renal function, lipid metabolism, glycated hemoglobin (HbA1c), and serum leptin basically amongst the study population and provide a guide on possible therapies and interventions for people with obesity and T2DM so that metabolic control can be improved and associated complications can be better managed or prevented. Our findings indicate that in diabetic non-obese patients and controls, there was no significant correlation between leptin and lipid profile parameters. However, a different picture emerged in diabetic obese patients, where a significant correlation was observed between leptin and triglyceride levels (*R* = 0.314, *P* < .05) as well as between leptin and LDL levels (*R* = 0.115, *P* < .05). There was no significant association between leptin and total cholesterol (*R* = 0.118, *P* > .05) or HDL (*R* = 0.123, *P* > .05) in diabetic obese patients. These findings align with previous studies that reported a positive correlation between leptin levels and triglyceride levels in obese populations.^[25,26]^ On the other hand, some studies have reported a negative correlation between leptin and HDL levels, highlighting the complex and conflicting relationship between leptin levels and lipid profiles.^[27,28]^

It is important to note that the conflicting results of previous studies may be due to differences in study populations, sample size, and other confounding factors. Further research is needed to clarify the relationship between leptin levels and lipid profiles in diabetic patients, particularly those with obesity. This will provide important insights into the mechanisms underlying this relationship and inform the development of effective strategies for managing lipid profiles in diabetic patients. The results of our study provide insights into the relationship between leptin levels and biophysical parameters in type 2 diabetes mellitus patients based on obesity status. Our findings indicate that in diabetic non-obese patients and controls, there was no significant correlation between leptin levels and any of the biophysical parameters measured. However, in diabetic obese patients, our results reveal a significant correlation between leptin levels and weight (*R* = 0.194, *P* < .05) as well as between leptin levels and BMI (*R* = 0.115, *P* < .05). Additionally, we found a significant correlation between the waist-hip ratio and leptin levels (*R* = 0.488, *P* < .05) in this group.

These findings are consistent with previous studies that reported a significant association between leptin levels and obesity-related parameters in individuals with type 2 diabetes mellitus.^[4,5]^ For instance, a study reported a positive correlation between leptin levels and waist circumference in Korean individuals with type 2 diabetes.^[7]^ Similarly, a study found a significant positive correlation between leptin levels and BMI in Saudi Arabian individuals with type 2 diabetes.^[9]^ These results suggest that obesity may play a role in the relationship between leptin levels and biophysical parameters in type 2 diabetes mellitus patients. Our study’s results indicated no statistically significant difference in urea, creatinine, or estimated glomerular filtration rate (eGFR) between diabetic obese and nondiabetic obese individuals. Conversely, a significant difference was observed in these parameters between the control group and the diabetic group. These findings align with previous studies that have established diabetes as a risk factor for decreased kidney function, as evidenced by elevated levels of creatinine and urea and a decrease in eGFR. For instance, a study found that patients with type 2 diabetes exhibited significantly lower eGFR compared to healthy controls.^[16]^ Similarly, a study reported that diabetes was linked with an increased risk of kidney dysfunction, indicated by elevated creatinine and urea levels.^[25]^ Our study results suggest that obesity, in the absence of diabetes, may not be a risk factor for impaired kidney function in patients with type 2 diabetes. This is a noteworthy discovery, as previous research has provided conflicting results on the impact of obesity on kidney function in people with diabetes.

Further studies are needed to understand the underlying mechanisms of the observed differences in kidney function between diabetic and nondiabetic obese individuals and assess the generalizability of these results to other populations. Also, further research is needed to clarify the mechanisms underlying this relationship and inform the development of effective strategies for managing type 2 diabetes mellitus in obese individuals. However, further research is needed to confirm these results and determine the underlying mechanisms responsible for the observed relationships between leptin, obesity, and biophysical parameters in type 2 diabetes mellitus patients. This could include exploring the role of insulin resistance and inflammation in mediating these relationships and evaluating the potential impact of other factors, such as diet and physical activity, on these relationships.

Strengths of this study include a well-defined patient population, standardized measures to assess lipid profile and glycemic control, and a large sample size that increases the statistical power of the results. However, there are also limitations that must be considered. First, the study was cross-sectional, so causality cannot be established. Second, the sample was drawn from a single center, so the generalizability of the results to other populations may be limited. Third, dietary and lifestyle factors that may impact lipid profile and glycemic control were not systematically assessed, and their potential impact on the results cannot be ruled out. The results should be interpreted in light of the study’s limitations, and future research is needed to investigate the underlying mechanisms further and determine the generalizability of the results to other populations.

## 5. Conclusion

The conclusions of our study add to the growing body of evidence that underscores the importance of weight management as a key component in the management of type 2 diabetes. Our results indicate that obesity has a negative impact on lipid profile and glycemic control in type 2 diabetes patients and highlight the significance of considering the effect of obesity on the relationship between leptin and lipid profile in diabetic patients. There was an observed correlation between serum leptin and dysregulation of lipid metabolism in individuals with type 2 diabetes and obesity, which is an indication that elevated serum leptin rate is a contributory factor to impaired metabolism of lipids with an observed increase in total cholesterol, HDL, and LDL in the study population. The study was also able to observe a significant correlation between serum leptin and HbA1c, making it responsible for impaired glycemic control as indicated by the rise in HbA1c values in individuals with type 2 diabetes and obesity, thus suggesting the potential role of serum leptin in impacting glucose metabolism and the onset and advancement of type 2 diabetes. With respect to renal function activity, an elevated serum leptin level can be attributed to impaired renal activity and an increased risk of diabetic nephropathy in individuals with type 2 diabetes. These findings suggest that targeting obesity and leptin levels may be an effective strategy for improving lipid profiles and glycemic control in type 2 diabetes. Also, the leptin signaling pathway could be targeted, or an approach to lower serum leptin levels might be a suitable drug intervention approach to manage and improve glycemic control, renal function, and lipid metabolism. Further research is needed to confirm these findings and explore the underlying mechanisms contributing to these differences.

One of the several limitations that our study faces is with respect to bias and confounding. It is important to note that the cross-sectional nature of the study makes it susceptible to selection bias, as participants may not be representative of the broader T2DM population. Additionally, the lack of randomization increases the risk of bias, as unmeasured factors may influence both exposure and outcome variables. Controlling for confounding variables is crucial in studies of this nature to identify the effects of variables of interest. However, despite efforts to adjust for potential confounders, residual confounding may persist due to unmeasured or inaccurately measured variables such as lifestyle factors like diet and physical activity, which influence both T2DM and the study variables, may not have been fully put into account.

Furthermore, the reliance on Pearson correlation analysis alone may oversimplify the complex relationships between variables and overlook nonlinear associations or interactions. While Pearson correlation assesses linear relationships between continuous variables, other statistical techniques such as multivariable regression could provide a more comprehensive understanding of the associations while controlling for confounders. Further research using more robust study designs and analytical methods is warranted to confirm these findings and elucidate the underlying mechanisms.

## Author contributions

**Conceptualization:** Akintunde Akintayo Akinjinmi.

**Data curation:** Akintunde Akintayo Akinjinmi.

**Formal analysis:** Akintunde Akintayo Akinjinmi.

**Investigation:** Akintunde Akintayo Akinjinmi.

**Methodology:** Akintunde Akintayo Akinjinmi, Adebayo Adetola Amballi, Abdulbasit Opeyemi Abdulrahman, Nicholas Aderinto, Akinbobola Ayokunle Adeniyi, Opeyemi Muili AbdulBasit, Emmanuel Ifeanyi Obeagu.

**Supervision:** Emmanuel Ifeanyi Obeagu.

**Validation:** Akintunde Akintayo Akinjinmi.

**Visualization:** Akintunde Akintayo Akinjinmi.

**Writing – original draft:** Akintunde Akintayo Akinjinmi, Adebayo Adetola Amballi, Abdulbasit Opeyemi Abdulrahman, Nicholas Aderinto, Akinbobola Ayokunle Adeniyi, Ibukun Akinwunmi Akindele, Opeyemi Muili AbdulBasit, Emmanuel Ifeanyi Obeagu.

**Writing – review & editing:** Akintunde Akintayo Akinjinmi, Adebayo Adetola Amballi, Abdulbasit Opeyemi Abdulrahman, Nicholas Aderinto, Akinbobola Ayokunle Adeniyi, Ibukun Akinwunmi Akindele, Opeyemi Muili AbdulBasit, Emmanuel Ifeanyi Obeagu.
